# A36 EFFECT OF FIBRE SUPPLEMENTATION IN CELIAC DISEASE: IMPLICATIONS FOR MUCOSAL HEALING

**DOI:** 10.1093/jcag/gwad061.036

**Published:** 2024-02-14

**Authors:** M Wulczynski, J Blom, G H Rueda, M Pinto-Sanchez, M Constante, S Rahmani, J Murray, D Armstrong, H Galipeau, E Verdu

**Affiliations:** . McMaster University Faculty of Health Sciences, Hamilton, ON, Canada; . McMaster University Faculty of Health Sciences, Hamilton, ON, Canada; . McMaster University Faculty of Health Sciences, Hamilton, ON, Canada; . McMaster University Faculty of Health Sciences, Hamilton, ON, Canada; . McMaster University Faculty of Health Sciences, Hamilton, ON, Canada; . McMaster University Faculty of Health Sciences, Hamilton, ON, Canada; . Mayo Clinic Division of Gastroenterology and Hepatology, Rochester, MN; . McMaster University Faculty of Health Sciences, Hamilton, ON, Canada; . McMaster University Faculty of Health Sciences, Hamilton, ON, Canada; . McMaster University Faculty of Health Sciences, Hamilton, ON, Canada

## Abstract

**Background:**

Celiac disease (CeD) is a T-cell mediated enteropathy driven by gluten in genetically susceptible individuals. Mucosal inflammation persists for years even when treated with a gluten-free diet (GFD). Although patients may be advised to increase dietary fibre, some fibres are poorly tolerated leading, potentially, to suboptimal fibre intake and hampering dietary recommendations.

**Aims:**

Our project aims to investigate whether and how the supplementation of two fibre types with distinct functional profiles affects the recovery of gluten-immunopathology in a mouse model of gluten-sensitivity. We hypothesize that: (1) HylonVII, but not inulin, accelerates mucosal recovery by increasing microbial carbolytic activity and production of short-chain fatty acids (SCFA); (2) fibre intake is decreased in CeD patients, leading to decreased SCFA production.

**Methods:**

NOD-DQ8 mice were immunized to gliadin and then placed on a gluten-containing diet. Three weeks later, mice were returned to GFD supplemented with or without HylonVII or inulin to 15% total fibre, for 6, 10, or 12 weeks. Recovery from gluten-induced pathology was evaluated by counting CD3^+^ intraepithelial lymphocytes (IELs) in villi tips and measuring villus-to-crypt (V/C) ratios in the small intestine. Adult CeD patients (newly diagnosed n=6 and GFD-treated n=7) and healthy controls (n=5) completed a food frequency questionnaire (Victoria DQES v2) and provided fecal samples for evaluation of SCFA production. Fecal SCFA were measured with targeted single ion monitoring gas-chromatography/mass-spectrometry (Agilent) as markers of fibre metabolism.

**Results:**

In gluten immunized mice, 3 weeks of wheat diet led to increased CD3^+^ IEL counts and decreased V/C ratios (*p*ampersand:003C0.01), which remained abnormal after 6 weeks of GFD. Mice consuming GFD+inulin, but not HylonVII, normalized CD3^+^ IELs and V/C ratios, had higher fecal SCFAs (*p*ampersand:003C0.05) (primarily acetate), and a higher carbolytic-proteolytic metabolite ratio (*p*ampersand:003C0.05) compared with mice consuming a GFD alone for six weeks. Only 1/7 patients with CeD on GFD met the minimum recommendation for fibre intake (25 g/day, Health Canada), compared with 5/11 participants on gluten-containing diets. SCFA production was impaired in active but not in treated CeD patients (2563 vs 3744 μg/g, *p*ampersand:003C0.01).

**Conclusions:**

In gluten-immunized NOD-DQ8 mice, GFD+inulin accelerates mucosal recovery, which is associated with increased fecal SCFA. In our pilot clinical data, patients on a GFD did not meet the recommendation for fibre intake although they were capable of metabolizing fibre to produce SCFA that could facilitate mucosal recovery. Together these results suggest that CeD on GFD may benefit from increasing fibre, such as inulin, during the GFD.

**Funding Agencies:**

CIHRCeliac Canada

